# Anxiety, Depression, and Adverse Clinical Outcomes in Patients With Atrial Fibrillation Starting Warfarin: Cardiovascular Research Network WAVE Study

**DOI:** 10.1161/JAHA.117.007814

**Published:** 2018-04-14

**Authors:** Christine Baumgartner, Dongjie Fan, Margaret C. Fang, Daniel E. Singer, Daniel M. Witt, John R. Schmelzer, Marc S. Williams, Jerry H. Gurwitz, Sue Hee Sung, Alan S. Go

**Affiliations:** ^1^ Division of Hospital Medicine University of California, San Francisco San Francisco CA; ^2^ Departments of Epidemiology #x0026; Biostatistics, and Medicine University of California, San Francisco San Francisco CA; ^3^ Department of General Internal Medicine Inselspital Bern Bern University Hospital University of Bern Switzerland; ^4^ Division of Research Kaiser Permanente Northern California Oakland CA; ^5^ Division of General Internal Medicine Massachusetts General Hospital, and Harvard Medical School Boston MA; ^6^ Department of Pharmacotherapy University of Utah Salt Lake City UT; ^7^ Marshfield Clinic Research Institute Marshfield Clinic Marshfield WI; ^8^ Genomic Medicine Institute Weis Center for Research Geisinger Danville PA; ^9^ Division of Geriatric Medicine Department of Medicine University of Massachusetts Medical School Worcester MA; ^10^ Meyers Primary Care Institute Worcester MA

**Keywords:** anxiety, atrial fibrillation, bleeding, depression, stroke, warfarin, Atrial Fibrillation, Quality and Outcomes, Ischemic Stroke, Intracranial Hemorrhage, Anticoagulants

## Abstract

**Background:**

Anxiety and depression are associated with worse outcomes in several cardiovascular conditions, but it is unclear whether they affect outcomes in atrial fibrillation (AF). In a large diverse population of adults with AF, we evaluated the association of diagnosed anxiety and/or depression with stroke and bleeding outcomes.

**Methods and Results:**

The Cardiovascular Research Network WAVE (Community‐Based Control and Persistence of Warfarin Therapy and Associated Rates and Predictors of Adverse Clinical Events in Atrial Fibrillation and Venous Thromboembolism) Study included adults with AF newly starting warfarin between 2004 and 2007 within 5 health delivery systems in the United States. Diagnosed anxiety and depression and other patient characteristics were identified from electronic health records. We identified stroke and bleeding outcomes from hospitalization databases using validated *International Classification of Diseases, Ninth Revision* (*ICD‐9*), codes. We used multivariable Cox regression to assess the relation between anxiety and/or depression with outcomes after adjustment for stroke and bleeding risk factors. In 25 570 adults with AF initiating warfarin, 490 had an ischemic stroke or intracranial hemorrhage (1.52 events per 100 person‐years). In multivariable analyses, diagnosed anxiety was associated with a higher adjusted rate of combined ischemic stroke and intracranial hemorrhage (hazard ratio, 1.52; 95% confidence interval, 1.01–2.28). Results were not materially changed after additional adjustment for patient‐level percentage of time in therapeutic anticoagulation range on warfarin (hazard ratio, 1.56; 95% confidence interval, 1.03–2.36). In contrast, neither isolated depression nor combined depression and anxiety were significantly associated with outcomes.

**Conclusions:**

Diagnosed anxiety was independently associated with increased risk of combined ischemic stroke and intracranial hemorrhage in adults with AF initiating warfarin that was not explained by differences in risk factors or achieved anticoagulation quality.

**Clinical Perspective**

**What Is New?**

- The impact of anxiety and depression diagnoses on adverse outcomes in patients with atrial fibrillation anticoagulated with warfarin is unclear.
- In a large cohort of adults with atrial fibrillation newly starting warfarin, diagnosed anxiety was associated with an increased risk of combined ischemic stroke and intracranial hemorrhage during follow‐up, whereas depression or combined depression and anxiety was not.
- This risk increase was not mediated by anticoagulation quality.

**What Are the Clinical Implications?**

- Diagnosed anxiety might be useful for further stratification of stroke and bleeding risk in patients with atrial fibrillation who newly start warfarin.
- Future studies should investigate underlying mechanisms and assess whether different treatment approaches might relate to improved outcomes in these patients.

Anxiety and depression are common disorders in the United States, with a lifetime risk of 29% and 17%, respectively.^1^ Affected individuals are at increased risk of cardiovascular events and death.^2^, ^3^, ^4^ Atrial fibrillation (AF) is the most common clinically significant cardiac arrhythmia in adults, with an estimated lifetime risk of 37% after age 55 years,^5^ and contributes to significant excess morbidity and mortality.^6^, ^7^ Both anxiety and depression frequently coexist with AF,^8^ but it is unclear whether anxiety and depression are associated with differences in outcomes in people with AF.

A recent study has shown that patients with AF with mental health disorders, such as anxiety or psychosis, who are eligible for warfarin therapy are less likely to receive treatment than patients without mental health disorders. This is possibly because of providers’ concerns about noncompliance with a complicated medication regimen,^9^ suggesting an uncertainty among healthcare providers about the risk‐benefit profile of warfarin among these patients. Evidence on treatment outcomes related to warfarin in patients with mental health disorders is sparse, and understanding the impact of anxiety and depression on clinical outcomes in patients on anticoagulation treatment is crucial to improve their management by identifying those who might benefit from more intensive monitoring or alternative anticoagulation strategies.

Therefore, we examined the association between diagnosed anxiety or depression with adverse clinical outcomes in adults initiating warfarin therapy for AF.

## Methods

### Study Design and Setting

The WAVE (Community‐Based Control and Persistence of Warfarin Therapy and Associated Rates and Predictors of Adverse Clinical Events in Atrial Fibrillation and Venous Thromboembolism) project in the Cardiovascular Research Network^10^ was a retrospective cohort study of adults with AF who were enrolled in 1 of 5 large integrated healthcare systems in the United States: Kaiser Permanente Northern California, which served >3.2 million members in Northern California; Kaiser Permanente Colorado, which served >460 000 members in the metropolitan area of Denver, CO; Geisinger, which served >1 million patients (of whom ≈450 000 members were covered by the Geisinger Health Plan) in central and northeast Pennsylvania; Marshfield Clinic, which served >550 000 members in central and northwest Wisconsin; and Harvard Pilgrim, which served >1 million members in New England during the study period. These healthcare systems represent demographically and socioeconomically diverse community‐based populations in the specific geographic areas.^11^ Research divisions from these healthcare delivery systems each created site‐specific virtual data warehouses containing individual patient information from electronic medical records and administrative databases to promote interinstitutional research.^10^ The virtual data warehouse was the primary data source for identification of patients and characterization of covariates in our study, and mortality data were obtained from State Death files and the National Death Index.

The study was approved by the institutional review boards of the participating organizations. Because of the nature of the study, a waiver of informed consent was obtained.

If requested, a complete, cleaned, deidentified copy of the data set can be made available through the Cardiovascular Research Network. For information on the data elements, format, and security, see http://CVRN.org. The Cardiovascular Research Network has a standard approach for data sharing that includes review of requests by the project Steering Committee based on the following: (1) high scientific merit; (2) consistency with the overall goals and objectives of the parent study; (3) provision of adequate resources to effectively complete the project, including sufficient budget to cover costs of personnel and data acquisition; (4) the requisite expertise to meet the objectives of the project; and (5) inclusion of the parent study team member(s) as part of the investigative team.

### Cohort Assembly

Eligible adults had a diagnosis of AF or atrial flutter and were newly initiating warfarin therapy between January 1, 2004, and December 31, 2007. AF diagnoses were based on a primary hospital discharge diagnosis of AF (*International Classification of Diseases, Ninth Revision* [*ICD‐9*] code 427.31 or 427.32) or ≥2 outpatient diagnoses of AF found in health plan databases. Information on warfarin prescription was obtained from dispensing data found in health plan pharmacy databases. The index date of warfarin therapy was defined as new warfarin dispensing without prior evidence of receiving warfarin within 3 years before the index dispensing date. Time on warfarin was based on the number of days supplied per prescription and intervening international normalized ratio (INR) tests using a previously validated algorithm.^12^ We included adults who were ≥21 years at their index warfarin prescription date, had at least 1 outpatient INR measurement after the index date, and had ≥12 months of continuous health plan membership and pharmacy drug benefit before study entry to ensure minimum necessary information on patient characteristics. Individuals with any warfarin exposure in the 3 years before study entry were excluded.

### Primary Predictors: Diagnosed Anxiety and/or Depression

We considered anxiety and/or depression diagnoses that were present at entry. Study participants were considered to have prevalent anxiety or depression at baseline if they had ≥2 outpatient diagnoses within 12 months of each other or 1 inpatient primary diagnosis based on *ICD‐9* codes found in health plan databases. A diagnosis of anxiety was defined as the presence of any of the following *ICD‐9* codes: 300.0x, 300.2x, 300.3, 309.20, 309.21, 309.24, and 309.81. A diagnosis of depression was defined as any of the following *ICD‐9* codes: 296.2x, 296.3x, 296.82, 298.0, 300.4, 301.12, 309.0, 309.1, 309.28, and 311, as has been used in a previous study.^13^

### Outcomes

Follow‐up started at the first warfarin dispensing date and ended on the date of an outcome event, withdrawal from the health plan, termination of warfarin use, death not attributable to an outcome event, or end of the study on December 31, 2007, whichever came first. Clinical events potentially related to AF or warfarin treatment were chosen as outcomes. The primary outcome was the rate of combined ischemic stroke and/or intracranial hemorrhage (ICH), the 2 most influential factors driving net clinical benefit of anticoagulation for AF.^14^ We also examined as a secondary outcome hospitalizations for extracranial hemorrhage. Clinical outcomes were identified by searching computerized hospitalization and billing databases for relevant *ICD‐9* codes (Data S1), and only the first event of each type was recorded. The diagnosis of ischemic stroke (*ICD‐9* code 433.01, 433.11, 433.21, 433.31, 433.81, 433.91, 434.01, 434.11, 434.91, or 436) was based on a relevant code in the primary hospital discharge position and ICH on a primary or secondary hospital discharge diagnosis.^10^, ^15^ Hospitalizations for extracranial hemorrhage were identified by appropriate *ICD‐9* codes in the primary position. Because all patients in this cohort were enrolled in healthcare delivery systems, we were able to identify outcomes even when patients presented to institutions outside of the particular healthcare delivery network, because care provided outside of owned facilities is systematically tracked.

### Covariates

We used the virtual data warehouse to obtain information on age, sex, race, relevant medical history, comorbid conditions, and other cardiovascular risk factors at baseline using previously validated approaches based on *ICD‐9* diagnosis and procedure codes, Current Procedure Terminology procedure codes, laboratory records, and pharmacy records.^15^, ^16^

Anticoagulation quality was defined as the percentage of time in a therapeutic INR range of 2.0 to 3.0. It was calculated as the total time that the patient had an INR between 2.0 and 3.0, estimated using a modified linear interpolation between measured INR points, divided by the total time the patient was on warfarin.^17^ Baseline renal function using serum creatinine concentration values was obtained from health system laboratory databases and estimated by the glomerular filtration rate (mL/min per 1.73 m^2^) using the Chronic Kidney Disease Epidemiology Collaboration equation.^18^

We searched health plan pharmacy databases for dispensed prescriptions for anxiolytic medications (buspirone, alprazolam, chlordiazepoxide, clonazepam, clorazepate, diazepam, lorazepam, estazolam, halazepam, oxazepam, prazepam, and quazepam) and antidepressant medications available during the study period (amitriptyline, amoxapine, bupropion, citalopram, clomipramine, desipramine, doxepin, duloxetine, escitalopram, fluoxetine, fluvoxamine, imipramine, maprotiline, mirtazapine, nefazodone, nortriptyline, paroxetine, phenelzine, protriptyline, selegiline, sertraline, tranylcypromine, trazodone, trimipramine, and venlafaxine). We also searched for cardiovascular medications, including angiotensin‐converting enzyme inhibitors, aldosterone receptor antagonists, β blockers, digoxin, diuretics, nitrates, and statins.

### Statistical Analysis

All analyses were performed using SAS statistical software, version 9.3 (Cary, NC). Group differences for baseline characteristics in patients stratified by anxiety and/or depression diagnoses were compared using ANOVA test for continuous variables and the χ^2^ test for categorical variables. Multivariable extended Cox regression models using time‐updated covariates were used to assess the relations between diagnosed anxiety and/or depression and incident adverse outcomes, adjusting for patient demographic factors, baseline medical conditions, renal function, and hemoglobin. We then further adjusted the analyses for the percentage of time in therapeutic INR range over each patient's follow‐up period, and then performed an analysis adjusting for baseline anxiolytic and antidepressant as well as cardiovascular medication use. To assess whether results applied to the subgroup of patients without prior ischemic stroke, we added a sensitivity analysis restricted to patients without a history of stroke at baseline.

## Results

We identified 25 570 eligible adults with diagnosed AF initiating a new course of warfarin therapy (Figure). A total of 897 participants (3.5%) had a diagnosis of anxiety only, 2322 (9.1%) had a diagnosis of depression only, and 904 (3.5%) had combined anxiety and depression (Table 1). Mean age was 72.3 years, and 43.5% were women in the overall cohort. Patients with a combined diagnosis of depression and anxiety were more likely to have a history of myocardial infarction, unstable angina, or percutaneous coronary intervention, whereas those without anxiety or depression were more likely to have diagnosed hypertension, but less likely to be affected by dementia or chronic lung or liver disease compared with the other subgroups. The percentage of time in therapeutic INR range differed among the 4 groups. It was 57% in patients without depression or anxiety and those with anxiety only, whereas it was 52% and 53% in patients with depression and those with combined depression and anxiety, respectively. *P* value < 0.001; results not shown.

**Figure 1:**
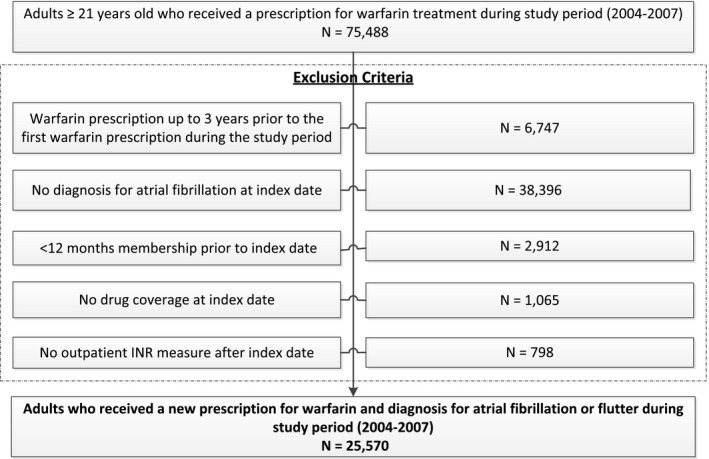
Selection of the final study population. INR indicates international normalized ratio.

**Table 1:** Characteristics of Study Participants at Baseline

Characteristic	No Anxiety or Depression (n=21 447)	Diagnosed Anxiety Only (n=897)	Diagnosed Depression Only (n=2322)	Diagnosed Anxiety and Depression (n=904)	*P* Value
Age, mean (SD), y	72.3 (11.2)	72.2 (11.5)	72.3 (10.8)	71.3 (11.5)	0.051
Female sex, n (%)	8711 (40.6)	554 (61.8)	1296 (55.8)	561 (62.1)	<0.001
Race, n (%)					0.005
White	16 946 (79.0)	727 (81.1)	1930 (83.1)	754 (83.4)	
Black	988 (4.6)	23 (2.6)	90 (3.9)	28 (3.1)	
Asian/Pacific Islander	1556 (7.2)	62 (6.9)	86 (3.7)	31 (3.4)	
Other	441 (2.1)	30 (5.2)	70 (3.0)	35 (3.9)	
Unknown	1516 (7.1)	55 (6.1)	146 (6.3)	56 (6.2)	
Medical history, n (%)					
Acute myocardial infarction	1141 (5.3)	47 (5.2)	163 (7.0)	68 (7.5)	0.008
Unstable angina	771 (3.6)	42 (4.7)	104 (4.5)	49 (5.4)	0.008
Coronary artery bypass surgery	766 (3.6)	20 (2.2)	89 (3.8)	28 (3.1)	0.47
Percutaneous coronary intervention	808 (3.8)	29 (3.2)	100 (4.3)	46 (5.1)	0.048
Ischemic stroke	1247 (5.8)	50 (5.6)	171 (7.4)	65 (7.2)	0.12
Transient ischemic attack	798 (3.7)	37 (4.1)	114 (4.9)	44 (4.9)	0.12
Other arterial thromboembolic event	188 (0.9)	7 (0.8)	30 (1.3)	10 (1.1)	0.55
Intracranial hemorrhage	123 (0.6)	4 (0.5)	24 (1.0)	7 (0.8)	0.54
Gastrointestinal hemorrhage	467 (2.2)	28 (3.1)	77 (3.3)	30 (3.3)	0.052
Other hospitalized bleed	138 (0.6)	8 (0.9)	17 (0.7)	9 (1.0)	0.23
Systemic cancer	3209 (15.0)	136 (15.2)	397 (17.1)	132 (14.6)	0.64
Chronic heart failure	4896 (22.8)	187 (20.9)	632 (27.2)	227 (25.1)	0.17
Diabetes mellitus	5408 (25.2)	195 (21.7)	741 (31.9)	230 (25.4)	0.85
Diagnosed hypertension	8050 (37.5)	302 (33.7)	757 (32.6)	282 (31.2)	<0.001
Diagnosed dementia	651 (3.0)	42 (4.7)	234 (10.1)	97 (10.7)	<0.001
Chronic lung disease	3877 (18.1)	187 (20.9)	533 (23.0)	198 (21.9)	0.014
Chronic liver disease	428 (2.0)	24 (2.7)	76 (3.3)	32 (3.5)	0.005
Dyslipidemia	7977 (37.2)	324 (36.1)	876 (37.7)	332 (36.7)	0.77
Mechanical fall	349 (1.6)	16 (1.8)	86 (3.7)	37 (4.1)	<0.001
Baseline eGFR (mL/min per 1.73 m^2^), n (%)					<0.001
≥60	13 104 (61.1)	594 (66.2)	1419 (61.1)	602 (66.6)	
30–59	6766 (31.5)	260 (29.0)	720 (31.0)	259 (28.7)	
<30 or dialysis	881 (4.1)	25 (2.8)	132 (5.7)	31 (3.4)	
eGFR missing	696 (3.3)	18 (2.0)	51 (2.2)	12 (1.3)	
Medication history, n (%)					
Antidepressant medication	2321 (10.8)	245 (27.3)	1442 (62.1)	606 (67.0)	<0.001
Antianxiety medication	1550 (7.2)	348 (38.8)	361 (15.6)	388 (42.9)	<0.001

eGFR indicates estimated glomerular filtration rate.

**Table 2:** Crude Outcome Event Rates in Adults on Warfarin for AF

Mental Health Condition	People, No.	Combined Ischemic Stroke and ICH		Ischemic Stroke		ICH		Hospitalization for Extracranial Hemorrhage	
		Events, No.	Event Rate per 100 Person‐Years	Events, No.	Event Rate per 100 Person‐Years	Events, No.	Event Rate per 100 Person‐Years	Events, No.	Event Rate per 100 Person‐Years
Overall	25 570	490	1.52	307	0.95	205	0.63	867	2.69
No anxiety, no depression	21 447	395	1.43	247	0.89	168	0.60	724	2.63
Anxiety only	897	24	2.32	16	1.54	10	0.96	32	3.10
Depression only	2322	54	2.06	30	1.14	24	0.91	87	3.31
Both anxiety and depression	904	17	1.74	14	1.43	3	0.30	24	2.44

AF indicates atrial fibrillation; and ICH, intracranial hemorrhage.

### Diagnosed Anxiety and/or Depression and Combined Ischemic Stroke and ICH

The median follow‐up was 339 days (interquartile range, 131–708 days), with a maximum follow‐up of 1460 days. In the overall cohort, 490 (1.9%) experienced an ischemic stroke or ICH event, for a rate of 1.52 events per 100 person‐years (Table 2). In multivariable adjusted analyses, diagnosed anxiety was associated with a significantly higher adjusted rate of combined ischemic stroke and ICH (hazard ratio, 1.52; 95% confidence interval, 1.01–2.28), whereas depression was not (Table 3). Additional adjustment for patient‐level time in therapeutic INR range did not materially affect the multivariable association between diagnosed anxiety and the risk of combined ischemic stroke and ICH (hazard ratio, 1.56; 95% confidence interval, 1.03–2.36). Further adjustment for the receipt of anxiolytic and antidepressant drugs or cardiovascular drugs did not lead to a meaningful change in the results (Table 3). In a sensitivity analysis with exclusion of patients with a history of ischemic stroke at baseline, a statistically significant association between diagnosed anxiety and combined ischemic stroke and ICH remained (adjusted hazard ratio, 1.56; 95% confidence interval, 1.03–2.36; Table S1).

**Table 3:** Serial Multivariable Analyses for the Association Between Anxiety and/or Depression and the Risk of Combined Ischemic Stroke and ICH in Adults With AF Initiating Warfarin Therapy

Mental Health Condition	Adjusted HR (95% CI)^a^	HR Additionally Adjusted for TTR (95% CI)	HR Additionally Adjusted for Use of Anxiolytic and Antidepressant Drugs (95% CI)^b^	HR Additionally Adjusted for Use of Cardiac Drugs (95% CI)^c^
No anxiety, no depression	Reference	Reference	Reference	Reference
Anxiety only	1.52 (1.01–2.28)	1.56 (1.03–2.36)	1.57 (1.01–2.43)	1.54 (0.99–2.38)
Depression only	1.20 (0.89–1.61)	1.14 (0.85–1.53)	1.07 (0.78–1.48)	1.08 (0.78–1.49)
Both anxiety and depression	0.93 (0.63–1.70)	0.98 (0.60–1.61)	0.95 (0.58–1.59)	0.96 (0.57–1.61)

AF indicates atrial fibrillation; CI, confidence interval; HR, hazard ratio; ICH, intracranial hemorrhage; and TTR, percentage of time in therapeutic international normalized ratio range.

a Adjusted by patient sex, baseline age, race, renal function, hemoglobin level, history of cancer, ischemic and hemorrhagic stroke, acute myocardial infarction, unstable angina, coronary artery bypass surgery, percutaneous coronary intervention, transient ischemic attack, intracranial hemorrhage, gastrointestinal bleeding, other major bleeding, other thromboembolic event, chronic heart failure, dementia, lung disease, liver disease, dyslipidemia, hypertension, mechanical fall, and diabetes mellitus.

b Adding baseline use of anxiolytic and antidepressant medications. Baseline definition is for drug prescribed 120 days before the index or at index date.

c Adding baseline cardiac drug use, including angiotensin‐converting enzyme inhibitors, aldosterone receptor antagonists, β blockers, digoxin, diuretics, nitrates, and statins. Baseline definition is for drug prescribed 120 days before the index until 90 days after index.

**Table 4:** Anxiety and/or Depression and the Risk of Ischemic Stroke, ICH, or Hospitalization for Extracranial Hemorrhage in Adults on Warfarin for AF

Mental Health Condition	Adjusted HR (95% CI)^a^		
	Ischemic Stroke	ICH	Hospitalization for Extracranial Hemorrhage
No anxiety, no depression	Reference	Reference	Reference
Anxiety only	1.59 (0.95–2.65)	1.63 (0.85–3.12)	1.17 (0.80–1.72)
Depression only	0.96 (0.65–1.41)	1.32 (0.84–2.06)	0.99 (0.78–1.27)
Both anxiety and depression	1.16 (0.66–2.03)	0.47 (0.15–1.47)	0.80 (0.52–1.25)

AF indicates atrial fibrillation; CI, confidence interval; HR, hazard ratio; and ICH, intracranial hemorrhage.

a Adjusted for patient sex, baseline age, race, renal function, hemoglobin level, percentage of time in therapeutic international normalized ratio range, history of cancer, ischemic and hemorrhagic stroke, acute myocardial infarction, unstable angina, coronary artery bypass surgery, percutaneous coronary intervention, transient ischemic attack, intracranial bleeding, gastrointestinal bleeding, other major bleeding, other thromboembolic event, chronic heart failure, dementia, lung disease, liver disease, dyslipidemia, hypertension, mechanical fall, and diabetes mellitus.

### Association Between Diagnosed Anxiety and/or Depression and Secondary Outcomes

Crude event rates of individual clinical outcomes are presented in Table 2. Diagnosed anxiety was associated with higher adjusted hazard ratios for ischemic stroke, ICH, and extracranial hemorrhage, but the associations were not statistically significant (Table 4). Neither diagnosed depression alone nor the combination of anxiety and depression was significantly associated with ischemic stroke, ICH, or hospitalization for extracranial hemorrhage (Table 4).

## Discussion

In a large, multicenter, community‐based cohort of adults newly treated with warfarin for AF, we found that having diagnosed anxiety was associated with an increased risk for worse outcomes independent of known risk factors and the quality of anticoagulation therapy. In contrast, diagnosed depression was not associated with an increased risk of adverse events in this population.

Other studies have noted that certain mental health conditions are associated with adverse cardiovascular outcomes,^4^, ^19^, ^20^ but this has only been evaluated in patients with AF in a limited number of studies. A recent retrospective cohort study among >100 000 patients on warfarin (≥65 000 with AF) from the Veterans Health Administration found that the risk of hemorrhage was slightly higher in those with mental health disorders (such as depression) compared with those without mental health disorders.^21^ However, this previous study included only patients who were taking warfarin for at least 6 months and likely represented a selected subset of patients who tolerated anticoagulation better.^22^ Another study using data from a Medicaid administrative claims database found an association between any psychiatric illness and an increased risk of thromboembolic and hemorrhagic events among patients with AF on warfarin, but did not specifically examine risk according to type of underlying condition.^23^

The mechanism behind the association of anxiety and adverse outcomes is not clear. In our study, we examined whether the increased risk of adverse events in patients with diagnosed anxiety may have been related to anticoagulation control, which is potentially modifiable. Anticoagulation quality depends on multiple factors, including age, sex, the targeted INR,^24^ the frequency of INR monitoring,^25^ medication adherence,^26^ diet, comorbidities,^24^ and drug‐drug interactions^27^; some of these factors may be negatively affected by mental health disorders.^28^, ^29^, ^30^ However, adjustment for patient‐level time in therapeutic INR range did not materially affect the strength of association for anxiety and outcomes. Information on certain behavioral factors (eg, smoking and physical inactivity)^31^ that may potentially confound the relationship between anxiety and outcomes was not consistently available and, thus, could not be analyzed. An intriguing avenue of further investigation may be how anxiety‐related stress can lead to alterations in hemostasis.^32^, ^33^ Autonomic arousal in the context of anxiety may result in physiologic changes, such as an increase in blood pressure,^34^ that are associated with the risk of both ischemic stroke and intracranial bleeding events.

We did not find a significant association between depression and adverse events in AF. Depression has been extensively studied as an independent risk factor for health outcomes, including cardiovascular disease and death,^2^, ^3^, ^35^ not only among the general population, but also in patients receiving anticoagulation.^36^, ^37^ Our study relied on *ICD‐9* codes to define anxiety and depression, and it is possible that a diagnosis of anxiety may be less sensitive for underlying anxiety disorders compared with depression diagnoses. Generalized anxiety disorder may be more underrecognized than major depression.^38^ A comparatively decreased sensitivity in anxiety diagnoses might lead to a selection of anxiety patients with higher disease severity, which could explain the increased risk of adverse outcomes in this population compared with patients with depression diagnoses or the combination of depression and anxiety.

A strength of our study is that we were able to include a large number of patients with diagnosed anxiety and depression from a geographically and ethnically diverse population of anticoagulated patients with AF. By including patients newly treated with warfarin, our study reflects outcomes from the start of treatment in real‐life clinical practice, where the choice of whether to anticoagulate a patient must be made without proven tolerance or patient adherence. We ascertained longitudinal warfarin exposure and assessed patient‐level time in therapeutic INR range, which allowed us to examine the potential impact of anticoagulation control on our results.

Our study also had limitations. Although there was a consistent pattern of an increased rate of stroke and bleeding outcomes in patients with diagnosed anxiety compared with those without anxiety or depression, our results did not reach statistical significance for the individual secondary outcomes (ischemic stroke, ICH, and hospitalization for extracranial hemorrhage), possibly because of insufficient power. As noted previously, anxiety disorders and depression are often underdiagnosed,^39^ which would likely lead to nondifferential misclassification that would bias our results towards the null. We also cannot exclude residual confounding by variables that are difficult to obtain from electronic health records, such as genetic factors, socioeconomic status and social network,^40^, ^41^ or stress,^32^ and we were not able to assess the role of physical inactivity or smoking in the association between anxiety or depression and adverse outcomes.^31^ Our study relied on anxiety and depression diagnosed in the medical record, which likely identified more severe cases. Finally, our results may not be completely generalizable to all populations and practice settings.

Given a consistent trend towards a higher risk of adverse events in patients with diagnosed anxiety, future research should investigate whether different treatment approaches might be associated with more favorable risk‐benefit profile in these patients. The increased risk of adverse events did not seem to be mediated by poor INR control, so more intensive INR monitoring might, therefore, not be sufficient to prevent adverse events in these patients. Whether anticoagulation with direct oral anticoagulants reduces the risk of adverse events in patients with anxiety remains to be examined. Additional studies are needed to confirm our findings and further explore underlying mechanisms and optimal management strategies.

In conclusion, diagnosed anxiety, but not depression, was linked to a higher rate of combined ischemic stroke and ICH in adults with AF initiating anticoagulation. This information may be helpful in further risk stratifying patients with AF.

## Author Contributions

Go, Fan, and Sung had full access to all data of the study and take responsibility for the integrity of the data and the accuracy of data analysis. Study concept and design: Go. Data acquisition: Go, Gurwitz, Singer, Williams, Witt, Schmelzer. Data analysis and interpretation: Baumgartner, Fan, Fang, Go, Sung. Drafting the article Baumgartner, Fang, Go. Critical revision of the article for important intellectual content: Baumgartner, Fan, Fang, Go, Gurwitz, Singer, Sung, Williams, Witt, Schmelzer. Statistical analyses: Fan. Secured funding: Go. Administrative, technical, or material support: Fan, Go, Sung. Study supervision: Go.

## Sources of Funding

This study was funded by the National Institute on Aging (R01 AG15478), the National Heart, Lung, and Blood Institute (U19 HL91179 and RC2HL101589), the National Institute of Mental Health (3U19HL91179‐4S1), and the National Institute on Aging (R24 AG045050). Baumgartner received support from the Swiss National Science Foundation (P2BEP3_165409).

## Disclosures

Singer receives research support from Boehringer Ingelheim and Bristol‐Myers Squibb; and serves as a consultant for Boehringer Ingelheim, Bristol‐Myers Squibb, Merck, Johnson and Johnson, Medtronic, and Pfizer. Go has received a research grant through his institution from iRhythm Technologies. The remaining authors have no disclosures to report.

## Supporting information


**Data S1.** Supplemental methods. *International Classification of Diseases, 9th Edition* (*ICD‐9*) Codes Used for the Definition of Outcomes of Interest.
**Table S1.** Main and Sensitivity Analysis for the Association Between Anxiety and/or Depression and the Risk of Combined Ischemic Stroke and Intracranial Hemorrhage in Adults With Atrial Fibrillation Initiating Warfarin Therapy

## References

[1] Kessler RC , Berglund P , Demler O , Jin R , Merikangas KR , Walters EE . Lifetime prevalence and age‐of‐onset distributions of DSM‐IV disorders in the National Comorbidity Survey Replication. Arch Gen Psychiatry. 2005;62:593–602. 15939837 10.1001/archpsyc.62.6.593

[2] Barth J , Schumacher M , Herrmann‐Lingen C . Depression as a risk factor for mortality in patients with coronary heart disease: a meta‐analysis. Psychosom Med. 2004;66:802–813. 15564343 10.1097/01.psy.0000146332.53619.b2

[3] Whooley MA . Depression and cardiovascular disease: healing the broken‐hearted. JAMA. 2006;295:2874–2881. 16804154 10.1001/jama.295.24.2874 PMC2771193

[4] Roest AM , Martens EJ , de Jonge P , Denollet J . Anxiety and risk of incident coronary heart disease: a meta‐analysis. J Am Coll Cardiol. 2010;56:38–46. 20620715 10.1016/j.jacc.2010.03.034

[5] Weng LC , Preis SR , Hulme OL , Larson MG , Choi SH , Wang B , Trinquart L , McManus DD , Staerk L , Lin H , Lunetta KL , Ellinor PT , Benjamin EJ , Lubitz SA . Genetic predisposition, clinical risk factor burden, and lifetime risk of atrial fibrillation. Circulation. 2017 Available at: http://circ.ahajournals.org/content/early/2017/11/10/CIRCULATIONAHA.117.031431. Accessed March 7, 2018. 10.1161/CIRCULATIONAHA.117.031431 PMC5840011 29129827

[6] Chugh SS , Havmoeller R , Narayanan K , Singh D , Rienstra M , Benjamin EJ , Gillum RF , Kim YH , McAnulty JH Jr , Zheng ZJ , Forouzanfar MH , Naghavi M , Mensah GA , Ezzati M , Murray CJ . Worldwide epidemiology of atrial fibrillation: a Global Burden of Disease 2010 Study. Circulation. 2014;129:837–847. 24345399 10.1161/CIRCULATIONAHA.113.005119 PMC4151302

[7] Global, regional, and national age‐sex specific all‐cause and cause‐specific mortality for 240 causes of death, 1990–2013: a systematic analysis for the Global Burden of Disease Study 2013. Lancet. 2015;385:117–171. 25530442 10.1016/S0140-6736(14)61682-2 PMC4340604

[8] Patel D , Mc Conkey ND , Sohaney R , Mc Neil A , Jedrzejczyk A , Armaganijan L . A systematic review of depression and anxiety in patients with atrial fibrillation: the mind‐heart link. Cardiovasc Psychiatry Neurol. 2013;2013:159850. 23710335 10.1155/2013/159850 PMC3655604

[9] Schmitt SK , Turakhia MP , Phibbs CS , Moos RH , Berlowitz D , Heidenreich P , Chiu VY , Go AS , Friedman SA , Than CT , Frayne SM . Anticoagulation in atrial fibrillation: impact of mental illness. Am J Manag Care. 2015;21:e609–e617. 26735294

[10] Magid DJ , Gurwitz JH , Rumsfeld JS , Go AS . Creating a research data network for cardiovascular disease: the CVRN. Expert Rev Cardiovasc Ther. 2008;6:1043–1045. 18793105 10.1586/14779072.6.8.1043

[11] Go AS , Magid DJ , Wells B , Sung SH , Cassidy‐Bushrow AE , Greenlee RT , Langer RD , Lieu TA , Margolis KL , Masoudi FA , McNeal CJ , Murata GH , Newton KM , Novotny R , Reynolds K , Roblin DW , Smith DH , Vupputuri S , White RE , Olson J , Rumsfeld JS , Gurwitz JH . The Cardiovascular Research Network: a new paradigm for cardiovascular quality and outcomes research. Circ Cardiovasc Qual Outcomes. 2008;1:138–147. 20031802 10.1161/CIRCOUTCOMES.108.801654

[12] Go AS , Hylek EM , Chang Y , Phillips KA , Henault LE , Capra AM , Jensvold NG , Selby JV , Singer DE . Anticoagulation therapy for stroke prevention in atrial fibrillation: how well do randomized trials translate into clinical practice? JAMA. 2003;290:2685–2692. 14645310 10.1001/jama.290.20.2685

[13] Croen LA , Zerbo O , Qian Y , Massolo ML , Rich S , Sidney S , Kripke C . The health status of adults on the autism spectrum. Autism. 2015;19:814–823. 25911091 10.1177/1362361315577517

[14] Singer DE , Chang Y , Fang MC , Borowsky LH , Pomernacki NK , Udaltsova N , Go AS . The net clinical benefit of warfarin anticoagulation in atrial fibrillation. Ann Intern Med. 2009;151:297–305. 19721017 10.7326/0003-4819-151-5-200909010-00003 PMC2777526

[15] Go AS , Hylek EM , Borowsky LH , Phillips KA , Selby JV , Singer DE . Warfarin use among ambulatory patients with nonvalvular atrial fibrillation: the anticoagulation and risk factors in atrial fibrillation (ATRIA) study. Ann Intern Med. 1999;131:927–934. 10610643 10.7326/0003-4819-131-12-199912210-00004

[16] Go AS , Chertow GM , Fan D , McCulloch CE , Hsu CY . Chronic kidney disease and the risks of death, cardiovascular events, and hospitalization. N Engl J Med. 2004;351:1296–1305. 15385656 10.1056/NEJMoa041031

[17] Rosendaal FR , Cannegieter SC , van der Meer FJ , Briet E . A method to determine the optimal intensity of oral anticoagulant therapy. Thromb Haemost. 1993;69:236–239. 8470047

[18] Levey AS , Stevens LA , Schmid CH , Zhang YL , Castro AF III , Feldman HI , Kusek JW , Eggers P , Van Lente F , Greene T , Coresh J ; CKD‐EPI (Chronic Kidney Disease Epidemiology Collaboration) . A new equation to estimate glomerular filtration rate. Ann Intern Med. 2009;150:604–612. 19414839 10.7326/0003-4819-150-9-200905050-00006 PMC2763564

[19] Shibeshi WA , Young‐Xu Y , Blatt CM . Anxiety worsens prognosis in patients with coronary artery disease. J Am Coll Cardiol. 2007;49:2021–2027. 17512358 10.1016/j.jacc.2007.03.007

[20] Kawachi I , Sparrow D , Vokonas PS , Weiss ST . Symptoms of anxiety and risk of coronary heart disease: the Normative Aging Study. Circulation. 1994;90:2225–2229. 7955177 10.1161/01.cir.90.5.2225

[21] Paradise HT , Berlowitz DR , Ozonoff A , Miller DR , Hylek EM , Ash AS , Jasuja GK , Zhao S , Reisman JI , Rose AJ . Outcomes of anticoagulation therapy in patients with mental health conditions. J Gen Intern Med. 2014;29:855–861. 24549520 10.1007/s11606-014-2784-2 PMC4026501

[22] Gupta A . Capsule commentary on Paradise et al., outcomes of anticoagulation therapy in patients with mental health conditions. J Gen Intern Med. 2014;29:892. 24595423 10.1007/s11606-014-2798-9 PMC4026498

[23] Schauer DP , Moomaw CJ , Wess M , Webb T , Eckman MH . Psychosocial risk factors for adverse outcomes in patients with nonvalvular atrial fibrillation receiving warfarin. J Gen Intern Med. 2005;20:1114–1119. 16423100 10.1111/j.1525-1497.2005.0242.x PMC1490282

[24] Witt DM , Delate T , Clark NP , Martell C , Tran T , Crowther MA , Garcia DA , Ageno W , Hylek EM , Warped C . Twelve‐month outcomes and predictors of very stable INR control in prevalent warfarin users. J Thromb Haemost. 2010;8:744–749. 20398186 10.1111/j.1538-7836.2010.03756.x

[25] Pokorney SD , Simon DN , Thomas L , Fonarow GC , Kowey PR , Chang P , Singer DE , Ansell J , Blanco RG , Gersh B , Mahaffey KW , Hylek EM , Go AS , Piccini JP , Peterson ED ; Outcomes Registry for Better Informed Treatment of Atrial Fibrillation I nvestigators. Patients’ time in therapeutic range on warfarin among US patients with atrial fibrillation: results from ORBIT‐AF registry. Am Heart J. 2015;170:141–148, 148.e141. 26093875 10.1016/j.ahj.2015.03.017

[26] Davis NJ , Billett HH , Cohen HW , Arnsten JH . Impact of adherence, knowledge, and quality of life on anticoagulation control. Ann Pharmacother. 2005;39:632–636. 15713790 10.1345/aph.1E464

[27] Hirsh J , Dalen J , Anderson DR , Poller L , Bussey H , Ansell J , Deykin D . Oral anticoagulants: mechanism of action, clinical effectiveness, and optimal therapeutic range. Chest. 2001;119:8s–21s. 11157640 10.1378/chest.119.1_suppl.8s

[28] Cramer JA , Rosenheck R . Compliance with medication regimens for mental and physical disorders. Psychiatr Serv. 1998;49:196–201. 9575004 10.1176/ps.49.2.196

[29] Osterberg L , Blaschke T . Adherence to medication. N Engl J Med. 2005;353:487–497. 16079372 10.1056/NEJMra050100

[30] Goldman L , Freidin R , Cook EF , Eigner J , Grich P . A multivariate approach to the prediction of no‐show behavior in a primary care center. Arch Intern Med. 1982;142:563–567. 7065791

[31] Bonnet F , Irving K , Terra JL , Nony P , Berthezene F , Moulin P . Anxiety and depression are associated with unhealthy lifestyle in patients at risk of cardiovascular disease. Atherosclerosis. 2005;178:339–344. 15694943 10.1016/j.atherosclerosis.2004.08.035

[32] Austin AW , Wissmann T , von Kanel R . Stress and hemostasis: an update. Semin Thromb Hemost. 2013;39:902–912. 24114007 10.1055/s-0033-1357487

[33] Krummenacher R , Lukas PS , Demarmels Biasiutti F , Begre S , Znoj H , von Kanel R . Relationship between psychological distress and endogenous anticoagulants in patients with a previous venous thromboembolic event. Clin Appl Thromb Hemost. 2011;17:171–180. 20308230 10.1177/1076029609354331

[34] Player MS , Peterson LE . Anxiety disorders, hypertension, and cardiovascular risk: a review. Int J Psychiatry Med. 2011;41:365–377. 22238841 10.2190/PM.41.4.f

[35] Lichtman JH , Froelicher ES , Blumenthal JA , Carney RM , Doering LV , Frasure‐Smith N , Freedland KE , Jaffe AS , Leifheit‐Limson EC , Sheps DS , Vaccarino V , Wulsin L . Depression as a risk factor for poor prognosis among patients with acute coronary syndrome: systematic review and recommendations: a scientific statement from the American Heart Association. Circulation. 2014;129:1350–1369. 24566200 10.1161/CIR.0000000000000019

[36] Michal M , Prochaska JH , Keller K , Gobel S , Coldewey M , Ullmann A , Schulz A , Lamparter H , Munzel T , Reiner I , Beutel ME , Wild PS . Symptoms of depression and anxiety predict mortality in patients undergoing oral anticoagulation: results from the thrombEVAL study program. Int J Cardiol. 2015;187:614–619. 25863736 10.1016/j.ijcard.2015.03.374

[37] Michal M , Prochaska JH , Ullmann A , Keller K , Gobel S , Coldewey M , Munzel T , Wiltink J , Beutel ME , Wild PS . Relevance of depression for anticoagulation management in a routine medical care setting: results from the thrombEVAL study program. J Thromb Haemost. 2014;12:2024–2033. 25292317 10.1111/jth.12743

[38] Wittchen HU , Kessler RC , Beesdo K , Krause P , Hofler M , Hoyer J . Generalized anxiety and depression in primary care: prevalence, recognition, and management. J Clin Psychiatry. 2002;63(suppl 8):24–34. 12044105

[39] Kasper S . Anxiety disorders: under‐diagnosed and insufficiently treated. Int J Psychiatry Clin Pract. 2006;10(suppl 1):3–9. 10.1080/13651500600552297 24931537

[40] Frasure‐Smith N , Lesperance F , Habra M , Talajic M , Khairy P , Dorian P , Roy D . Elevated depression symptoms predict long‐term cardiovascular mortality in patients with atrial fibrillation and heart failure. Circulation. 2009;120:134–140, 133p following 140 19564557 10.1161/CIRCULATIONAHA.109.851675

[41] Lukas PS , Neugebauer A , Schnyder S , Biasiutti FD , Krummenacher R , Ferrari ML , von Kanel R . Depressive symptoms, perceived social support, and prothrombotic measures in patients with venous thromboembolism. Thromb Res. 2012;130:374–380. 22560851 10.1016/j.thromres.2012.04.011

