# Supplementary material for: Anxiety, Depression, and Adverse Clinical Outcomes in Patients With Atrial Fibrillation Starting Warfarin: Cardiovascular Research Network WAVE Study
Source: J Am Heart Assoc. 2018 Apr 14;7(8):e007814. doi: 10.1161/JAHA.117.007814 (PMC6015441; doi:10.1161/JAHA.117.007814)
Supplement: Supplementary file 1 — Data S1. Supplemental methods. International Classification of Diseases, 9th Edition (ICD‐9) Codes Used for the Definition of Outcomes of Interest. Table S1. Main and Sensitivity Analysis for the Association Between Anxiety and/or Depression and the Risk of Combined Ischemic Stroke and Intracranial Hemorrhage in Adults With Atrial Fibrillation Initiating Warfarin Therapy [file JAH3-7-e007814-s001.pdf]

SUPPLEMENTAL MATERIAL

Data S1.

Supplemental Methods. International Classification of Diseases, 9th Edition (ICD-9) Codes Used for the Definition of Outcomes of Interest

Ischemic Stroke:

Principal hospital discharge diagnosis of following ICD-9 codes:

433.01, 433.11, 433.21, 433.31, 433.81, 433.91, 434.01, 434.11, 434.91, 436.

Intracranial Hemorrhage:

Primary or secondary hospital discharge diagnosis of following ICD-9 codes:

430, 431, 432, 432.0, 432.1, 432.9, 852, 852.0, 852.00, 852.01, 852.02, 852.03, 852.04, 852.05, 852.06, 852.09, 852.1, 852.10, 852.11, 852.12, 852.13, 852.14, 852.15, 852.16, 852.19, 852.2, 852.20, 852.21, 852.22, 852.23, 852.24, 852.25, 852.26, 852.29, 852.3, 852.30, 852.31, 852.31, 852.33, 852.34, 852.35, 852.36, 852.39, 852.4, 852.40, 852.41, 852.42, 852.43, 852.44, 852.45, 852.46, 852.49, 852.5, 852.50, 852.51, 852.52, 852.53, 852.54, 852.55, 852.56, 852.59, 853, 853.0, 853.00, 853.01, 853.02, 853.03, 853.04, 853.05, 853.06, 853.09, 853.1, 853.10, 853.11, 853.12, 853.13, 853.14, 853.15, 853.16, 853.19.

Hospitalization for Extracranial Hemorrhage

ICD-9 codes for gastrointestinal bleeding or other bleeding in the primary position:

455.2, 455.5, 455.8, 456.0, 456.20, 530.7, 530.82, 531.00, 531.01, 531.20, 531.21, 531.40, 531.41, 531.60, 531.61, 532.00, 532.01, 532.20, 532.21, 532.40, 532.41, 532.60, 532.61, 533.00, 533.01, 533.20, 533.21, 533.40, 533.41, 533.60, 533.61, 534.00, 534.01, 534.20, 534.21, 534.40, 534.41, 534.60, 534.61, 535.01, 535.11, 535.21, 535.31, 535.41, 535.51,

535.61, 537.83, 562.02, 562.03, 562.12, 562.13, 568.81, 569.3, 569.85, 578.0, 578.1, 578.9,

423.0, 599.7, 719.11, 784.7, 784.8, 786.3, 287.8, 287.9, 627.0 , 627.1 , 626.2, 998.11, 459.0.

Table S1.Main and Sensitivity Analysis for the Association between Anxiety and/or Depression and the Risk of Combined Ischemic Stroke and Intracranial Hemorrhage in Adults with Atrial Fibrillation Initiating Warfarin Therapy

Mental Health Condition	Adjusted HR (95% CI)*	Adjusted HR (95% CI)* Restricted to Patients Without a History of Ischemic Stroke at Baseline
No anxiety, no depression	Reference	Reference
Anxiety only	**1.52 (1.01 - 2.28)**	**1.56 (1.03 – 2.36)**
Depression only	1.20 (0.89 - 1.61)	1.16 (0.87 – 1.55)
Both anxiety and depression	0.93 (0.63 - 1.70)	1.03 (0.63 – 1.68)

Abbreviations: CI, confidence interval; HR, hazard ratio.

*Adjusted by patient sex, baseline age, race, renal function, hemoglobin level, history of cancer, ischemic and hemorrhagic stroke, acute myocardial infarction, unstable angina, coronary artery bypass surgery, percutaneous coronary intervention, transient ischemic attack, intracranial hemorrhage, gastrointestinal bleeding, other major bleeding, other thromboembolic event, chronic heart failure, dementia, lung disease, liver disease, dyslipidemia, hypertension, mechanical fall, diabetes mellitus.
